# Cell surface‐anchored syndecan‐1 ameliorates intestinal inflammation and neutrophil transmigration in ulcerative colitis

**DOI:** 10.1111/jcmm.12934

**Published:** 2016-08-25

**Authors:** Yan Zhang, Zhongqiu Wang, Jun Liu, Shaoheng Zhang, Jiaxi Fei, Jing Li, Ting Zhang, Jide Wang, Pyong W. Park, Ye Chen

**Affiliations:** ^1^ State Key Laboratory of Organ Failure ResearchGuangdong Provincial Key Laboratory of GastroenterologyDepartment of GastroenterologyNanfang HospitalSouthern Medical UniversityGuangzhouGuangdongChina; ^2^ Department of Radiation Oncology and Cyberknife CenterKey Laboratory of Cancer Prevention and TherapyTianjin Medical University Cancer Institute & HospitalNational Clinical Research Center for CancerTianjinChina; ^3^ Department of GastroenterologyLiuzhou Worker's HospitalLiuzhouChina; ^4^ Department of GastroenterologyZhujiang HospitalSouthern Medical UniversityGuangzhouChina; ^5^ Department of MedicineChildren's HospitalHarvard Medical SchoolBostonMAUSA

**Keywords:** ectodomain shedding, intestinal inflammation, NF‐κB pathway, neutrophil transmigration, pro‐inflammatory cytokine, syndecan‐1

## Abstract

Syndecan‐1 (SDC1), with a variable ectodomain carrying heparan sulphate (HS) chains between different Syndecans, participates in many steps of inflammatory responses. In the process of proteolysis, the HS chains of the complete extracellular domain can be shed from the cell surface, by which they can mediate most of SDC1's function. However, the exact impact on SDC1 which anchored on the cell surface has not been clearly reported. In our study, we established the models by transfection with the cleavable resistant SDC1 mutant plasmid, in which SDC1 shedding can be suppressed during stimulation. Role of membrane SDC1 in inflammatory pathway, pro‐inflammatory cytokine secretion as well as neutrophil transmigration, and how suppressing its shedding will benefit colitis were further investigated. We found that the patients suffered ulcerative colitis had high serum SDC1 levels,presented with increased levels of P65, tumour necrosis factor alpha (TNF‐α) and IL‐1β and higher circulating neutrophils. NF‐κB pathway was activated, and secretion of TNF‐α, interleukin‐1beta (IL‐1β), IL‐6 and IL‐8 were increased upon lipopolysaccharide stimuli in intestinal epithelial cells. Syndecan‐1, *via* its anchored ectodomain, significantly lessened these up‐regulation extents. It also functioned in inhibiting transmigration of neutrophils by decreasing CXCL‐1 secretion. Moreover, SDC1 ameliorated colitis activity and improved histological disturbances of colon in mice. Taken together, we conclude that suppression of SDC1 shedding from intestinal epithelial cells relieves severity of intestinal inflammation and neutrophil transmigration by inactivating key inflammatory regulators NF‐κB, and down‐regulating pro‐inflammatory cytokine expressions. These indicated that compenstion and shedding suppression of cytomembrane SDC1 might be the optional therapy for intestinal inflammation.

## Introduction

Ulcerative colitis (UC) is a worldwide, chronic, idiopathic and inflammatory disease of the rectal and colonic mucosa. A combination of environmental factors and genetic predisposition seems to cause the disease, but to date, the definitive mechanism is still unknown 1. Although a variety of endoscopic, radiological and histological criteria are recommended, the difficulties in differential diagnosis and easy recurrence of UC are always challenging 2.

Syndecan‐1 (CD138, SDC1), a member of the syndecan family of heparan sulphate (HS) proteoglycans, is predominantly expressed on the basolateral membrane of epithelial cells. Syndecan‐1 is mainly identified with a long variable ectodomain between different Syndecan members, a short conserved cytoplasmic domain and a transmembrane domain 3. The ectodomain of SDC1 carries heparin sulphate chains which acts as a ligand‐binding mediate for most of its function 4. Through the HS chain, SDC1 can bind a variety of molecules, including cytokines, chemotactic factors, extracellular matrix components and even heparin‐binding proteins on the bacterial surface. Thereby, it plays critical roles in matrix remodelling, tissue repair, regulation of immune function and resolution of inflammation 5, 6.

Recently, studies have demonstrated that SDC1 participates in the composition of tight junction and maintains the function of the mucosa barrier. Syndecan‐1 could restore the dysfunction of ZO‐1 and occludin in cell monolayers by activating Stat3, maintain the epithelial integrity, protect enterocytes during interactions with bacteria and lessen epithelial permeability 7. The extracellular domain of SDC1, replete with the HS chains, can be proteolytically released from the cell surface by a process known as ectodomain shedding 8. This process is activated in response to injury or infection, and occurs as a part of the host's regulated responses to inflammation, microbial pathogenesis and wound healing 9. For example, in injured mouse corneas, SDC1 shedding is induced by *Staphylococcus aureus* virulence factors. And inhibition of shedding attenuates bacterial virulence 10. In acute lung injury mouse model, knockout of matrix metalloproteinase 7 (a protein that functions in defence, repair and inflammation) resulted in a lack of shed SDC1 in the alveolar fluid, and confining neutrophils in the interstitium of injured lungs 11. Moreover, SDC1 also has a protective effect during experimental colitis. Syndecan‐1‐null mice showed a significantly increased lethality, impaired mucosal healing and prolonged recruitment of inflammatory cells; while treatments with functional SDC1 ectodomain analogue significantly improved the symptoms 12. Therefore, SDC1 plays critical roles in the resolution of inflammation. However, the precise mechanism is not known clearly enough.

Neutrophil transmigration and accumulation in luminal spaces is a hallmark of mucosal inflammation. Neutrophils migrate out of the circulation, across both endothelial and epithelial barriers in acute inflammatory responses, which result in the pathogenesis of inflammatory disorders, failure of pathogen clearance and worsening severity 13. In intestine, neutrophil accumulation and abscess formation at the epithelial surface are pathological features of inflammatory disease processes including UC, infectious colitis and necrotizing enterocolitis 14. The pathological contributions from neutrophils are essential during these inflammation, whereas only a few of the molecular interactions related are known 15. Current studies have found that activated Sdc1 shedding in lung, liver and kidney of endotoxemic mice was associated closely with the resolution of accumulated neutrophils by removing sequestered CXC chemokines, while in SDC1‐null mice, neutrophilic inflammation was exaggerated, leading to organ damage and lethality 16. In SDC1‐deficient mice colitis model, neutrophils acted increasing adhesion to endothelial cells and intercellular adhesion molecule‐1(ICAM‐1), which increased leukocyte influx leading to an overshooting inflammatory reaction, as well as failing to resolve the leukocyte infiltrate and potentially increase lethality 12. In spite of these, interactions among SDC1, neutrophil transmigration and inflammatory responses are still rarely investigated, and the potential roles of SDC1 extracellular domain in inflammatory process of UC have not been fully explored.

In this study, we aimed to identify the role of epithelial‐expressed SDC1 in neutrophilic inflammation and neutrophil transmigration. The potential role of SDC1 in pro‐inflammatory cytokine secretion and inflammatory pathway was elucidated *in vitro* and *in vivo*. The association between SDC1 shedding and neutrophil transmigration in intestinal cells unravels a novel mechanism underlying the contribution of SDC1 to intestinal inflammation, and suggests that the use of SDC1 ectodomain could have a particular therapeutic impact on intestinal neutrophilic inflammation.

## Materials and methods

### Reagents and antibodies

The RPMI‐1640 or DMEM medium and fetal bovine serum (FBS) were purchased from Gibco, Inc. (Carlsbad, CA, USA). Phorbol 12‐myristate 13‐acetate (PMA) and lipopolysaccharide (LPS) from *Escherichia coli* (serotype 0111:B4) were obtained from Sigma‐Aldrich (St. Louis, MO, USA). Goat antimouse SDC1 antibody, and human tumour necrosis factor alpha (TNF‐α, Cat. DTA00C), interleukin‐1beta (IL‐1β, Cat. DLB50), cytokine‐induced neutrophil chemoattractant ELISA kits (CINC‐1, CXCL‐1, Cat. QC101) were all purchased from R&D Systems, Inc. (Minneapolis, MN, USA). NF‐κB P65 (8242) and NF‐κB p65 (3033) antibody were purchased from Cell Signaling Technology Inc. (Danvers, MA, USA). Mouse anti‐human SDC1 antibody and CD15 antibody was purchased from Santa Cruz Biotechnology Inc. (Dallas, TX ,USA) and ZSGB‐Bio Inc. (Beijing, China) respectively. Human Sdc1 ELISA kit was purchased from Elabscience Biotechnology Co., Ltd (Cat. E‐EL‐H1298; Wuhan, China).

### Tissue samples

Formalin‐fixed, paraffin‐embedded tissue samples from 20 patients with UC and 20 normal controls were randomly obtained on endoscopic examination from the Department of Gastroenterology, Nanfang Hospital during 2011–2012, and had been processed by routine clinical histopathological methods. Definitive diagnosis of UC was established by standard endoscopic, histological and clinical criteria 17. The partial Mayo score was determined previously as described and classified into remission stage (less than two) and active stage(more than two) 18. Patients with septic complications, short bowel syndrome or cancer, and pregnant women were excluded. Their sociodemographic and clinical data are summarized in Table 1.

**Table 1:** Baseline demographic and clinical features of the study population

	UC		Healthy control
	Active	Remission	
Patients	16	4	20
Gender(male/female)	8/6	3/3	11/9
Median age in years(IQR)	44.5 (28–52.5)	41 (25.25–53.25)	38.5 (29.75–49)
Median partial Mayo score(IQR)	8 (6.25–11)	0.5 (0–1.75)	
Disease extent			
Proctitis	3	1	
Left‐sided colitis	2	1	
Extensive colitis	11	2	
Medications			
Treated patients (*n*)	13	4	
5‐ASA	13	4	
Steroids	9	2	
AZA	7	1	
Anti‐TNF‐α agents	11	4	

UC: ulcerative colitis; ASA: aminosalicylic acid; AZA: azathioprine; TNF‐a: tumour necrosis factor alpha; IQR: interquartile range.

Venous blood was collected after an overnight fast. Routine blood tests were analysed soon. Serum was obtained by centrifugation at 1800 g for 10 min., and the aliquots of the serum were stored at −80°C until analysis. The study was carried out in accordance with the institutional ethical guidelines and had been approved by the medical ethics committee of Southern Medical University. Informed consent was obtained from all patients.

### Immunohistochemical analysis and evaluation

The immunohistochemical methods have been described previously 19. Sections were deparaffinized, rehydrated, quenched of endogenous peroxidase, antigen retrieved and incubated with the indicated primary antibodies diluted by 1:100 overnight at 4°C. After extensively washing, slides were incubated with a secondary reagent, developed with the DAB reagent (ZSGB‐Bio Inc, Beijing, China), counterstained with haematoxylin and mounted. Negative controls were incubated without antibody or non‐specific rat IgG at an equivalent protein concentration. The histological specimens were examined by two senior pathologists blinded to the protocol. The score was determined according to the intensity of staining independently, as ranked from grade 0 to 3: 0, less than 10% of cells stained; 1, 10–50% of cells stained; 2, 50–75% of cells stained; 3, more than 75% of cells stained. For SDC1, only the membranes of surface epithelial cells and glandular epithelium cells with staining were scored. Meantime, tissue specimens were stained with haematoxylin and eosin and classified as normal mucosa, mild, moderate or severe enteritis (score 0–3), according to the proportion of sample crypt cross‐sections containing intraluminal or intra‐epithelial neutrophils.

### Construction of the SDC1 expression plasmid

A full‐length mouse SDC1 cDNA corresponding to bases 1–3057 of Genbank accession no. NM_011519.2 was amplified by PCR from a normal intestinal cDNA library (extracted from normal newborn BALB/c mouse). The primers for SDC1 were 5′‐GTGAAGCTTATGAGACGCGCGGCGCTCTG‐3′ (forward) and 5′‐GTGGATCCTCAGGCGTAGAACTCCTCCTGCTTG‐3′ (reverse). The PCR product was cloned into the pcDNA3.0 vector (Invitrogen, Carlsbad, CA, USA) to create the pcDNA3.0‐wild‐type SDC1 plasmid (wt‐SDC1). A mutant form of SDC1 which stably anchored at cell surface in response to shaddase, such as PMA and LPS, was constructed (cleavable‐resistant SDC1 mutant, mut‐SDC1). Site‐directed mutations at positions 243 and 244 (where the cleavage site of SDC1 was first found) 20 were carried out by overlap extension PCR using four specific primers: (*i*) 5′‐GTGAAGCTTATGAGACGCGCGGCGCTCTG‐3′, (*ii*) 5′‐CTTCCTGTCCAAAAGGCTCTGCCAAGTACCTGTGGCTCCTTCGTCC‐3′, (*iii*) 5′‐GGAGCCACAGGTACTTGGCAGAGCCTTTTGGACAGGAAGGAAGTGC‐3′, (*iv*) 5′‐GTGGATCCTCAGGCGTAGAACTCCTCCTGCTTG‐3′. The orientation of the insert and the sequence of cDNA were verified by sequencing, and the recombinant plasmids were transfected into cells with use of Lipofectamine 2000 (Invitrogen, Carlsbad, CA, USA) respectively.

### Cell culture and treatments

The intestinal epithelial cell lines including HT29, SW620, SW480, LoVo, HCT116, Caco‐2 and IEC‐6 were purchased from American Type Culture Collection, and cultured routinely in RPMI 1640 (HT29, SW620, SW480, LoVo and HCT116) or DMEM (Caco‐2 and IEC‐6) medium respectively, supplemented with 10% or 20% FBS at 37°C in 5% CO_2_. Cultured or transfected cells were incubated with 1 μM PMA for 15 min. or 1 μg/ml LPS from *E. coli* for 24 hrs, then processed as follows.

### Reverse transcription‐PCR

Total RNA was extracted from cells using Trizol (Invitrogen, CA, USA). RNA samples were subjected to RT using a First Strand cDNA Synthesis Kit (Takara, Dalian, China). The primers used are listed in Table 2. PCR was initiated by a 5‐min. incubation at 94°C, and ended after a 10‐min. extension at 72°C, with 40 cycles for denaturation at 94°C for 30 sec., annealing at 60°C for 30 sec., and extension at 72°C for 1 min. using a PCR kit (SBS Gene Tech Co., Beijing, China). GAPDH mRNA was amplified simultaneously as an internal control.

**Table 2:** Primers used for PCR

Gene	Forward primer	Reverse primer
Human		
TNF‐α	AAGTGGACCTTAGGCCTTCC	AGCCTATTGTTCAGCTCCGT
IL‐1β	CAGGACAGTCAGCTCTCTCC	ATGTGGGAGCGAATGACAGA
IL‐8	GACCCAACCACAAATGCCAG	CTGACCAGAAGAAGGAATGCC
IL‐6	GTCTGTTGTAGGGTTGCCAG	GGATATTCTCTTGGCCCTTGG
Rat^a^		
TNF‐α	CAAAGGCGGAGATGAGACCC	AGGCTTCTCCTTTGTGGTGAG
IL‐1β	TCCATTCCTGAGAGCCAAGC	TTAGCATGCCTGCCCTGAAC
IL‐6	CAGCTGATGCTGCCTATTGC	GGACGCACTCACCTCTTGTT
GAPDH	CACATCGCTCAGACACCATG	TGACGGTGCCATGGAATTTG

a The mRNA level of IL‐8 in IEC‐6 cells was not determined because there is no orthologue for IL‐8 gene in rat.

### Western blot and dot‐blot assay

To detect the expression of cell surface SDC1 and components of the inflammatory pathway, cells were lysed in RIPA (Radio Immunoprecipitation Assay) buffer with 1% PMSF, protease inhibitor cocktail and phosphatase inhibitor. Protein was loaded onto an SDS‐PAGE minigel and transferred onto a PVDF membrane. The blot was probed with primary antibody (1:1000) at 4°C overnight, washed and incubated with the appropriate HRP‐conjugated secondary antibody. Signals were visualized using ECL Substrates (Millipore, Boston, MA, USA). GAPDH was used as an endogenous protein for normalization.

To detect the shed SDC1 ectodomain, cell culture supernatant was applied to a ABS‐Tween buffer (50 mM sodium acetate NaOAc, 150 mM sodiurn chloride, 0.1% Tween20) moistened PVDF membrane under a mild vacuum, in a dot‐blot apparatus (Whatman, Maidstone, Kent, England). After three washes with PBS buffer, the membrane was incubated overnight in an anti‐SDC1 antibody (1:1000) at 4°C, followed by incubation with secondary antibody, and was then exposed to ECL substrates.

### Immunofluorescence assay

Cells were fixed in iced methanol for 10 min., blocked for 1 hr at room temperature in PBS containing 5% bovine serum albumin, and incubated at 4°C overnight with an anti‐SDC1 antibody (1:100). Cells without antibody incubation were used as control. After three washes, cells were labelled with PE‐conjugated IgG for 1 hr at room temperature, stained with DAPI for 10 min., mounted on glass slides, and examined with a fluorescence microscope.

### ELISA

Concentrations of SDC1, TNF‐α, IL‐1β and CXCL‐1 in the cell culture supernatant or serum samples were determined by sandwich‐type ELISA, performed according to the manufacturer's instructions. Absorbance was read at 450 nm, and the concentration was determined by comparing their optical densities to a standard curve.

### Isolation of neutrophils and migration assay

Five or two millilitres of venous blood from healthy people or from rats, respectively, was drawn into heparin‐containing collection tubes and processed within 2 hrs. Neutrophils were isolated as described previously and cell viability were evaluated by trypan blue staining 21. For the migration assay, 0.5 × 10^6^ neutrophils in 10% FBS medium were added to the upper insert of each transwell polyester membrane filter (6.5‐mm‐diameter inserts, 3.0‐μm pore size; BD, NY, USA) and 500 μl cell culture supernatant was collected from the epithelial cells at the indicated time‐point (when secretion of CXCL‐1 was highest), and added to the matched lower chamber. After 24 hrs incubation, non‐migrated cells were removed from the upper surface with a cotton swab. Cells that had migrated to the lower membrane surface were fixed in methanol and stained with 0.1% crystal violet, and cells in the lower supernatant were counted by trypan blue staining. Migration rate was calculated as a percentage of total neutrophils added to the upper insert.

### Dextran sodium sulphate‐induced colitis models

Dextran sulphate sodium salt (DSS) (MW: 36,000–50,000 Da; MP Biomedicals, Santa Ana, CA, USA) was used to induce colitis in mice, as described previously 22. A total of 40 male BALB/c mice (6–8 weeks old, 20 ± 2 g) were obtained from the Animal Center of Southern Medical University, China. The experimental protocol was approved by the Institutional Animal Care and Use Committee of Southern Medical University, China.

All animals were housed four per cage under specific pathogen‐free conditions (room temperature‐controlled at 24 ± 2°C with a relative humidity of 60 ± 5%, on a 12 hrs light/dark cycles) and fed *ad lib* with standard rodent chow and fresh normal saline. After a 1‐week quarantine, mice were randomly divided into five treatment groups: fresh distilled water control, DSS, DSS plus vector, DSS plus wt‐SDC1 and DSS plus mut‐SDC1. All mice, except for those in the control group, were given 4% DSS for 6 days. On day 7, mice in the DSS plus vector, DSS plus wt‐SDC1 and DSS plus mut‐SDC1 groups were given an intra‐peritoneal injection of *in vivo*‐jet PEI (Polyplus, Illkirch, France)‐mediated indicated DNA transfection twice (once every 3 days) respectively. The DSS group was injected with PBS. The control group was given fresh distilled water for drink and an injection of the same volume of PBS. All mice were given fresh distilled water for drink for another 7 days, and were killed on day 14.

During DSS colitis, the disease activity index (DAI) was determined by scoring the extent of bw loss (ranking 0–4, 0: no weight loss; 1: weight loss within 5%; 2: within 5–10%; 3: within 11–15%; 4: more than 15%), stool hemoccult positivity or gross bleeding (0: normal; 2: guiac; 4: gross bleeding), and stool consistency (0: normal; 2: loose; 4: diarrhoea). The final DAI was equal to one‐third of the combined score. Histology of tissue from the colons, and mucosal expression of SDC1, P65, p‐P65 were studied by haematoxylin and eosin staining, immunohistochemistry or western blot respectively. The scores were all determined by an investigator who was blind to the experimental groups 7. Blood was collected, and ELISA used to measure serum levels of TNF‐α and IL‐1β.

### Statistical analysis

All data from independent experiments were expressed as mean ± S.E.M, and were processed using SPSS 13.0 statistical software (IBM, Armonk, NY, USA). Experiments were repeated at least twice for assays. Student's *t*‐test (between two independent groups) and one‐way anova with Duncan multiple range test (among multiple independent groups) was used to compare differences in focused groups. Spearman's correlation analysis was used to define associations. The level of significance was defined as *P* < 0.05.

## Results

### Increased cellular SDC1 shedding and activated inflammatory factors were observed in patients with UC

**Figure 1:**
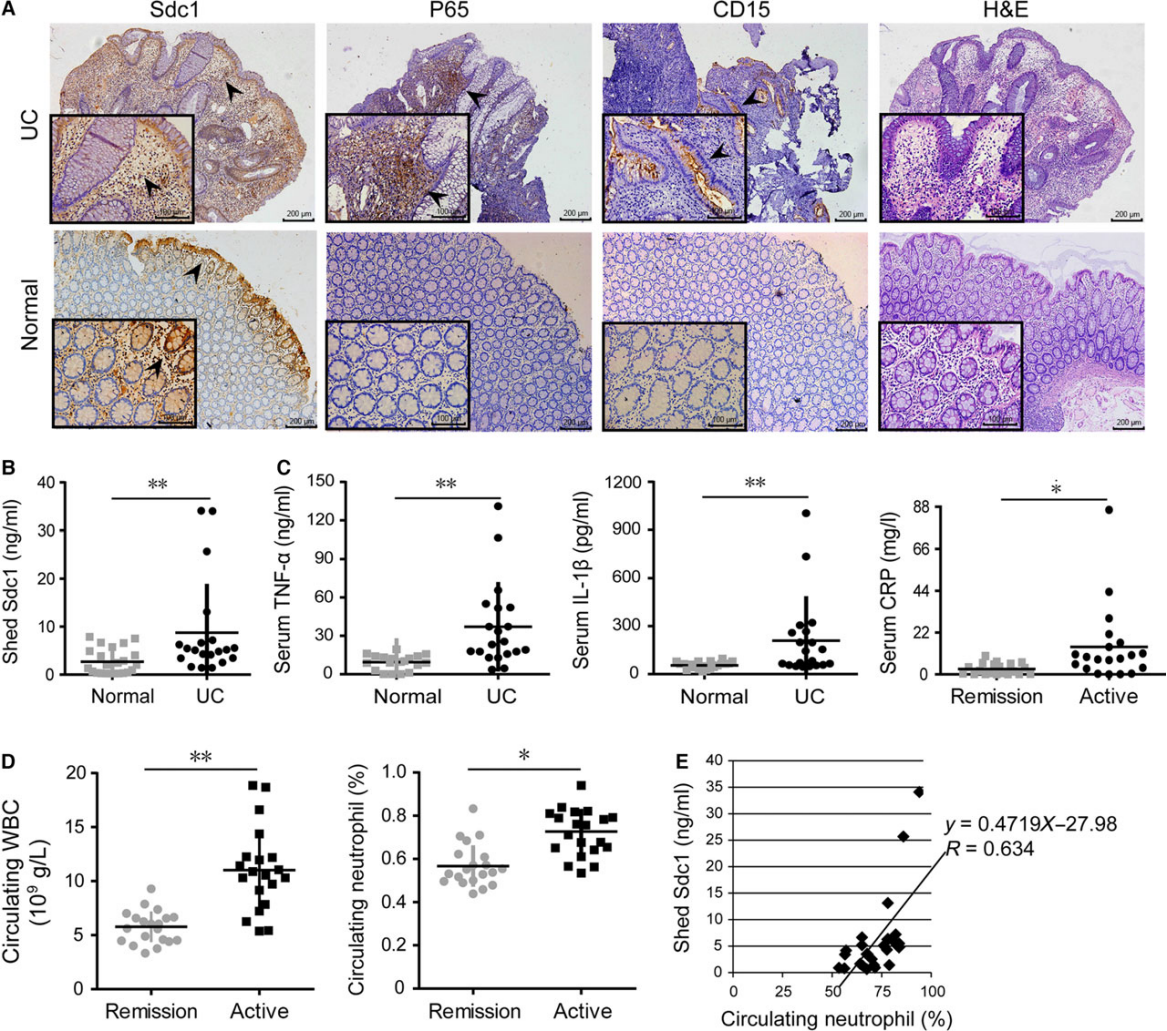
Levels of shed SDC1 and P65, secretion of TNF‐α and IL‐1β, as well as circulating neutrophil rate in ulcerative colitis patients. (**A**) Representative immunostaining of SDC1, P65 and CD15 in intestinal mucosa with overview (100×) and a magnification (400×). The right lane: haematoxylin and eosin staining. (**B**) Levels of shed SDC1 in serum was detected by ELISA;* n* = 20. (**C**) Levels of secreted TNF‐α (*n* = 20) and IL‐1β (*n* = 20) in serum were detected by ELISA; Circulating CRP was significantly higher in UC patients at active stage, *n* = 20. (**D**) Circulating white blood cells and neutrophils were significantly higher in UC patients at active stage; *n* = 20. (**E**) Shed SDC1 in serum was positively correlated with circulating neutrophil rate (*R* = 0.634, *P* < 0.01). *n* = 20. *P < 0.05, **P < 0.01.

In patients with UC, cellular SDC‐1 shed from the cell surface was significantly activated. Syndecan‐1 expresses on the cellular membrane of UC patients was lower than that of normal controls (Fig. 1A). Syndecan‐1 staining was present mainly in the membrane in normal mucosa, whereas its expression in UC patients was either barely detectable on cell membranes or mainly located in stromal cells. Meanwhile, the expression of soluble SDC‐1 in serum of UC patients was almost three times higher than in normal controls (*P* < 0.01, Fig. 1B), suggesting that the increased SDC‐1 shedding may because of the decrease in cell surface SDC‐1 located in colon mucosa.

Immunohistochemistry staining showed significantly positive expression of P65, a canonical component of NF‐κB pathway which was detected in intestinal epithelium of UC patients, while staining of P65 in normal mucosa was weak or absent (Fig. 1A). Parallelly, serum levels of pro‐inflammatory cytokines, TNF‐α and IL‐1β, were also significantly higher in UC patients than in normal controls (*P* < 0.01, Fig. 1C), same as CRP (*P* < 0.05, Fig. 1C), following a similar pattern as SDC1.

### Higher circulating neutrophils in UC patients positively correlate with activated serum SDC1

Because inflammatory factors, such as TNF‐α, IL‐1β and NF‐κB, are reported to be involved in the recruitment of several cell types of leucocyte 23, we tested whether the specific neutrophil subset, critical in pathogenesis of UC 24, responds to this activation. Circulating white blood cells and neutrophils were both elevated in UC patients in active stage compared with those in either patients in remission stage (*P* < 0.01, Fig. 1D) or normal controls (data not shown). Moreover, a scatterplot revealed a significantly positive correlation between shed SDC1 in serum and circulating neutrophil rate (*R* = 0.634, *P* < 0.01, Fig. 1E). Otherwise, immunohistochemistry staining and haematoxylin and eosin staining showed a high level of CD15 in colitic mucosa and intense inflammatory cell infiltration in lamina propria, both demonstrated more neutrophil recruitment into the intestine of UC patients (Fig. 1A).

### Shed SDC1 from cell surface was accompanied by activated inflammatory pathway and cytokine secretion in HT29 cells

**Figure 2:**
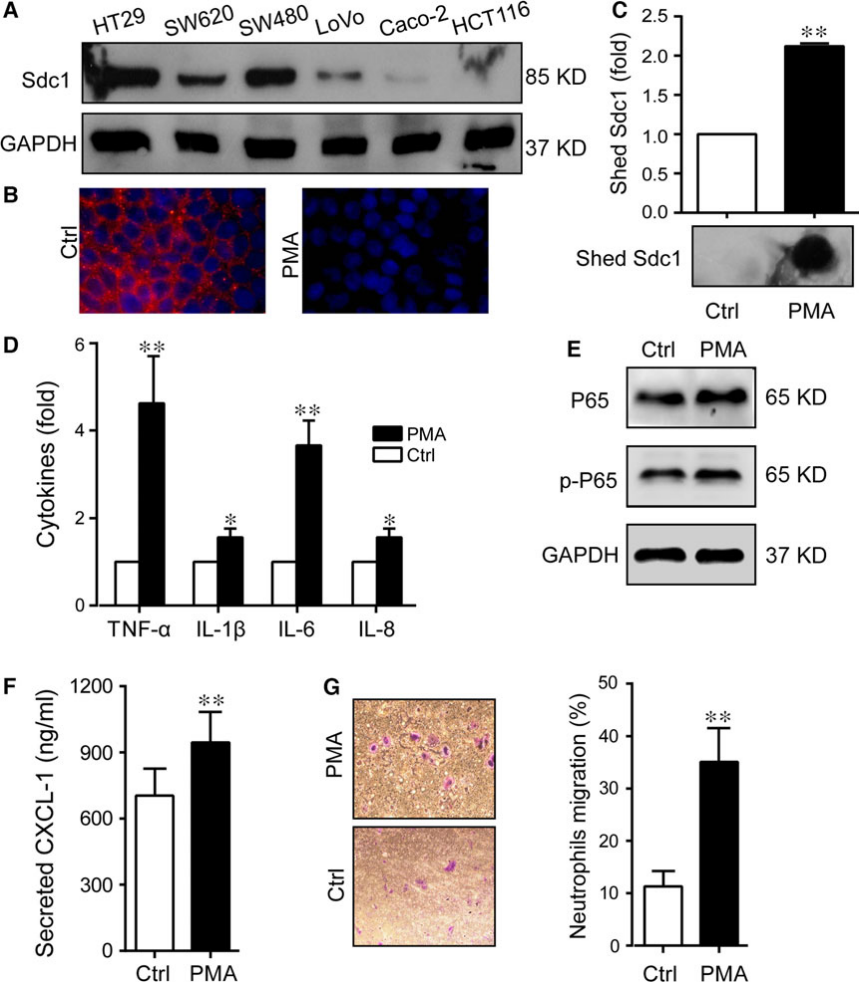
Shed SDC1 and inflammatory responses induced by PMA in HT29 cells. PMA was used to induce SDC1 shedding in HT29 cells. (**A**) Levels of Sdc1 in six intestinal epithelial cells were detected by western blot. GAPDH was used as the loading control. (**B**) Level of cell surface SDC1 was detected by immunofluorescence. SDC1 (red), cell nucleus (blue), original magnifications: 40×. (**C**) Levels of shed SDC1 in the cell culture supernatant were detected by ELISA (upper) and dot blot (lower). (**D**) Levels of secreted TNF‐α, IL‐1β, IL‐6 and IL‐8 in the cell culture supernatant were detected by PCR. (**E**) Levels of P65 and phosphorylated P65 were detected by Western blot. (**F**) Secretion of CXCL‐1 was detected by ELISA. (**G**) Neutrophils were isolated from human venous blood, and their transmigration was measured with use of transwell inserts. Crystal violet staining was used to observe the cell quantity change. Values represent mean ± S.E.M (*n* = 3), *P < 0.05, **P < 0.01, based on *t*‐test.

The SDC1 shedding from cell surface is induced by PMA, and results in the appearance of the free SDC1 ectodomain in the medium where the cells are growing 24. Thus, we used PMA to treat cultured cells and establish a cell model in which shed SDC1 is activated. We first examined the SDC1 expression in six colonic epithelial cell lines. Western blot showed that both HT29 and SW480 had prominent SDC1 expression, while Caco‐2 had much lower SDC1 expression, therefore HT29 cells were selected as a model here (Fig. 2A). Upon PMA stimulation, SDC1 on the cellular membrane decreased (Fig. 2B) and soluble SDC1 shed into the cell supernatant significantly increased detected by both dot blot and ELISA assay (*P* < 0.01, Fig. 2C). Resembling the increased level of shed SDC1, levels of secreted pro‐inflammatory cytokines including TNF‐α, IL‐1β, IL‐6 and IL‐8 were all elevated (*P* < 0.01, Fig. 2D). Moreover, inflammation‐associated NF‐κB pathway including P65 and p‐P65 were also activated in cells with activated SDC1 shedding (Fig. 2E).

### Shed SDC1 from cell surface was accompanied by increased CXCL‐1 secretion and neutrophil migration

Neutrophils are a vital component of the innate immune system, and are the first leucocytes that were recruited to the inflammatory site; migration of neutrophils is always preceded by generation of the many chemotactic factors, among which the most important ones are IL‐8 and CXCL‐1 15. Thus, we detected the level of CXCL‐1 in supernatants from HT29 cells treated by PMA. Enzyme‐linked immunosorbent assays showed that CXCL‐1 secretion displayed a similar pattern with shed soluble SDC1 upon PMA stimulation (Fig. 2F), suggesting that PMA can induce both SDC1 shedding and CXCL‐1 secretion. Then, we collected the cell growth media to treat neutrophils isolated from human venous blood, and found that migration of human neutrophils treated with the media from PMA‐stimulated cells (with higher level of IL‐8 and CXCL‐1) was significantly higher than the media from untreated cells (*P* < 0.01, Fig. 2G).

### Cell surface‐anchored SDC1 inhibited inflammatory pathway in Caco‐2 cell

**Figure 3:**
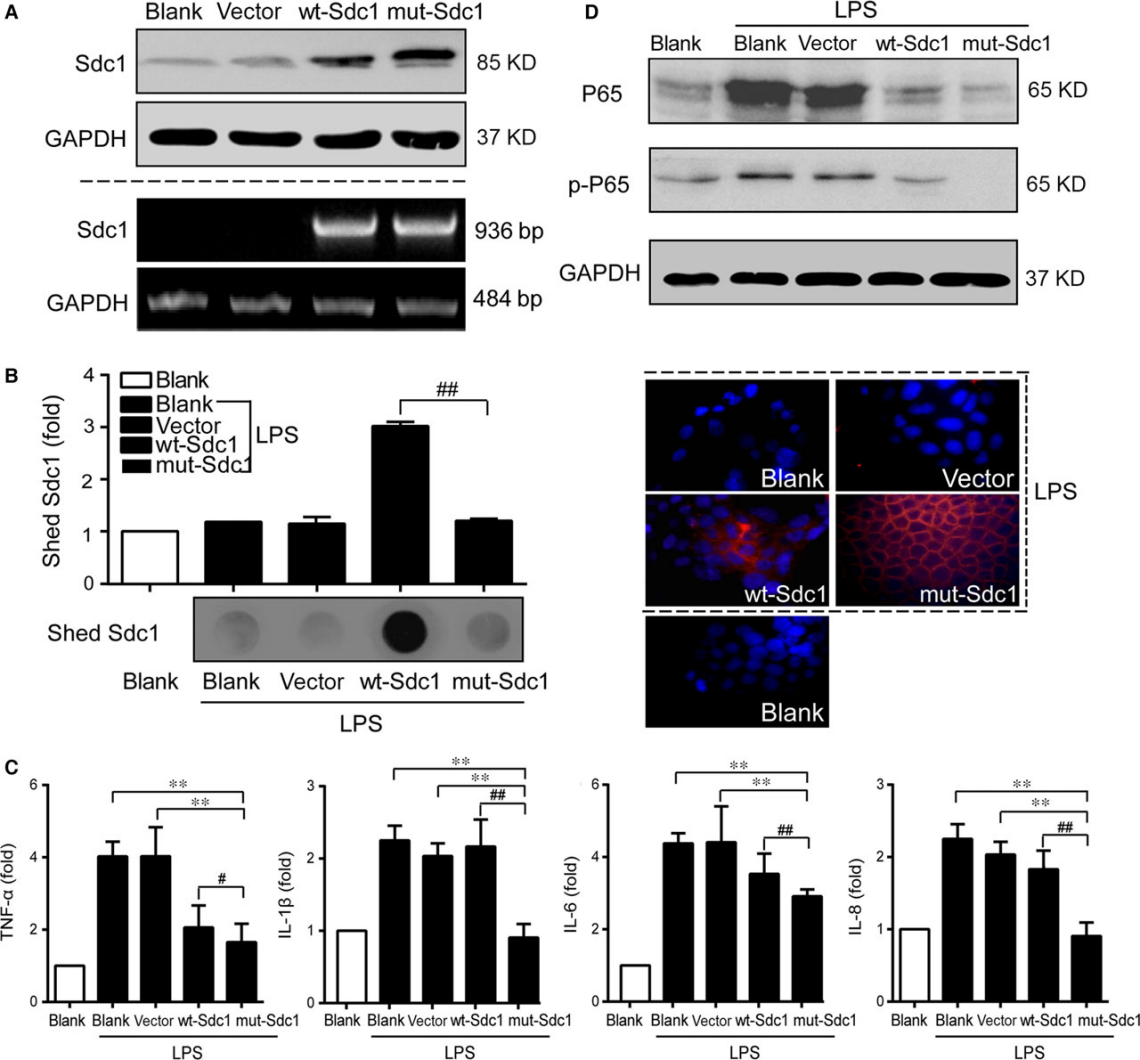
Cellular inflammatory responses induced by LPS was inhibited by cell surface‐anchored SDC1 in Caco‐2 cells. SDC1 shedding is blocked in Caco‐2 cells transfected with mut‐SDC1 plasmid and LPS was used to induce inflammatory responses. (**A**) Protein (upper) and mRNA (lower) levels of cell surface SDC1 were measured by western blot and PCR respectively. GAPDH was used as the loading control. (**B**) Levels of shed SDC1 in the cell culture supernatant were detected by ELISA (left upper) and dot blot (left lower). Protein level of cell surface SDC1 upon LPS stimulation was measured by immunofluorescence (right). SDC1 (red), cell nucleus (blue), original magnifications: 400×. (**C**) Cell surface‐anchored SDC1 down‐regulated secretion of TNF‐α, IL‐1β, IL‐6 and IL‐8 in the cell culture supernatant detected by PCR. (**D**) Cell surface‐anchored SDC1 inactivated NF‐κB pathway detected by Western blot. (**E**) Cell surface‐anchored SDC1 alleviated impaired viability induced by LPS, but had no significant effect on cell proliferation. Values represent mean ± S.E.M (*n* = 3) and were analysed by Duncan's multiple range test for multiple comparison in anova (**P < 0.01. # significance between wt‐SDC1 and mut‐SDC1, ##*P* < 0.01).

It is reported that PMA treatment alone might induce inflammation despite the function of SDC1 19. Thus, to further obtain direct evidence on the relationship between SDC1 and inflammation‐related factors, we establish a cell model in which no SDC1 shedding occurs subsequent to PMA stimulation by transfecting a mutant form of mut‐SDC1 plasmid into intestinal epithelial cells. At 24 hrs post transfection, we found marked increases in the expression of cell surface SDC1, both at the mRNA and protein levels, in Caco‐2 cells transfected with wt‐SDC1 or mut‐SDC1 plasmid, compared with the parental and vector‐transfected cells (Fig. 3A). Conditioned growth media was collected to measure levels of shed SDC1. After stimulation with LPS, dramatic increases were detected in the soluble ectodomains of SDC1 in the conditioned medium from wt‐SDC1‐transfected cells, whereas shed SDC1 from mut‐SDC1‐transfected cells was significantly suppressed (*P* < 0.01, Fig. 3B).

Lipopolysaccharide stimulation led to significant up‐regulation of TNF‐α, IL‐1β, IL‐6 and IL‐8 in Caco‐2 cells. Transfection with wt‐SDC1 and mut‐SDC1 led to down‐regulation of these cytokines, but the decrease was more notable in the mut‐SDC1‐transfected cells (*P* < 0.01, Fig. 3C). The NF‐κB pathway presented with induced phosphorylation and activation in response to LPS; but the induced activity was markedly less in cells transfected with mut‐SDC1(Fig. 3D). These data suggest that SDC1 negatively regulates the pro‐inflammatory cytokine secretion and inflammatory pathway activation, and this influence may be primarily dependent on the suppression of ectodomain shedding.

### Cell surface‐anchored SDC1 inhibited CXCL‐1 secretion and neutrophil migration

**Figure 4:**
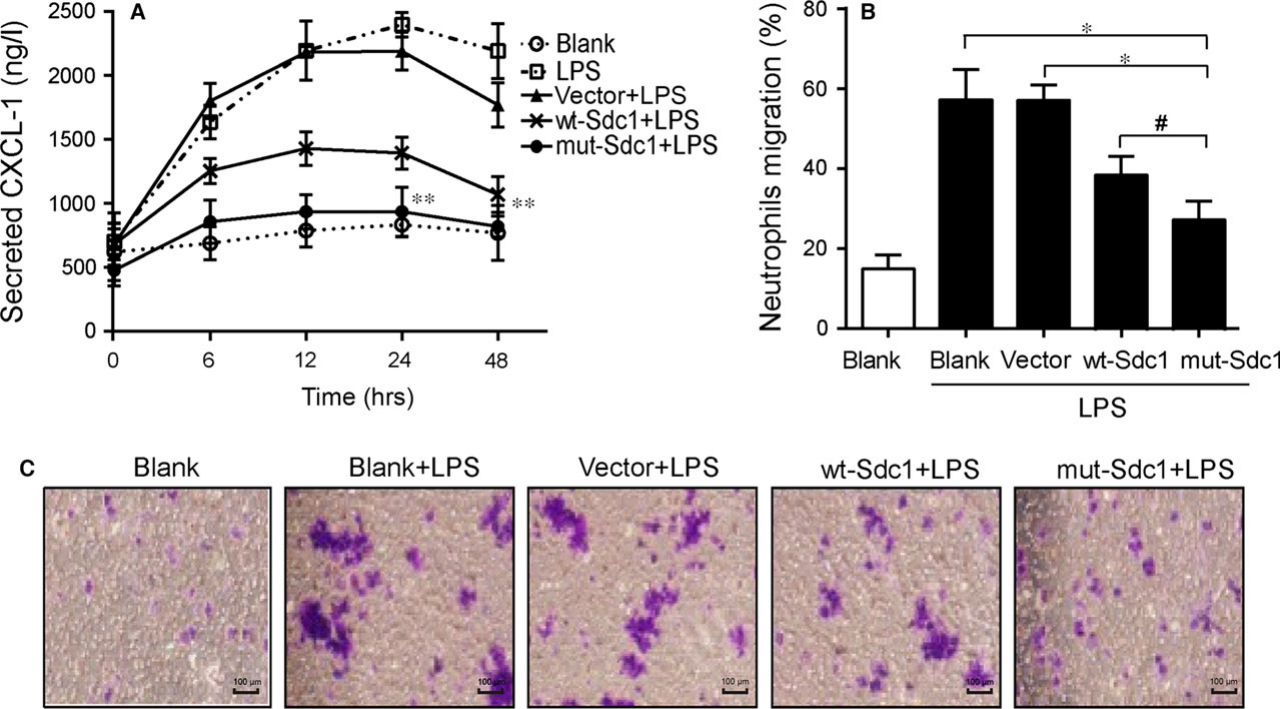
Secreted CXCL‐1 induced by LPS and sequent neutrophil transmigration was inhibited by cell surface‐anchored SDC1 in Caco‐2 cells. LPS was use to induce secretions of CXCL‐1, and expression of CXCL‐1 was assessed by ELISA. (**A**) The level of secreted CXCL‐1 was down‐regulated by Sdc1. (**B** and **C**) Cell culture supernatants containing high concentrations of CXCL‐1 were used to induce migration of human neutrophils. CXCL‐1 secretion promoted migration of neutrophils; this promoting effect was diminished when cells were transfected with mut‐SDC1. The transmigration was measured with use of transwell inserts, crystal violet staining was used to observe the cell quantity change. Values represent mean ± S.E.M (*n* = 3) and were analysed by Duncan's multiple range test for multiple comparison in anova (*P < 0.05, **P < 0.01. #significance between wt‐SDC1 and mut‐SDC1, #*P* < 0.05).

Use the previous model, we examined whether levels of cell surface SDC1 influence levels of CXCL‐1, and thus affected the transmigration of neutrophils. Following LPS stimulation, the levels of CXCL‐1 started increasing in supernatants from Caco‐2 cells, in a time‐dependent manner, followed by a slight decrease after 24 hrs. Syndecan‐1 inhibited the secretion of CXCL‐1, as seen in cells transfected with wt‐SDC1 or mut‐SDC1; this inhibitory effect was especially greater in the presence of cell surface anchored SDC1 (*P* < 0.01, Fig. 4A).

CXCL‐1 levels were highest at 24 hrs after LPS stimulation; thus we collected conditioned growth media at 24 hrs to treat neutrophils isolated from human venous blood using a transwell filter. Migration of neutrophils treated with media from LPS‐stimulated cells was significantly higher than when the media was from untreated cells. But this effect on migration was markedly inhibited when neutrophils were treated with media from wt‐SDC1‐ and mut‐SDC1‐transfected cells, with media from mut‐SDC1‐transfected cells (containing shed SDC1) mediating a more marked inhibition (*P* < 0.05, Fig. 4B and C).

### Cell surface‐anchored SDC1 ameliorates colitis activity, and mitigates the histological changes in the colon during DSS‐induced colitis

**Figure 5:**
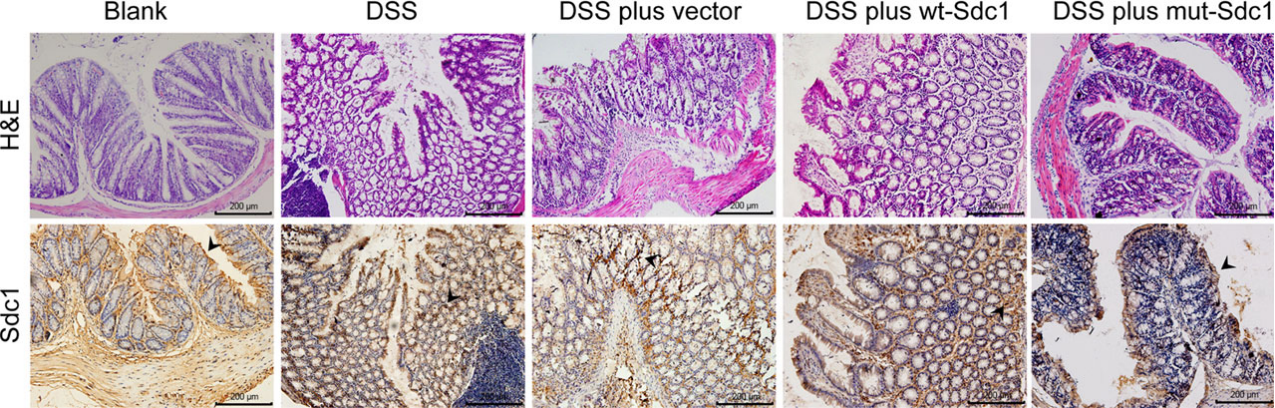
Effects of cell surface SDC1 on histology of colitis in mice. Immunohistochemical staining of mucosal SDC1 (lower) and haematoxylin and eosin staining (upper) in sections of mouse intestine. Original magnifications: 200×.

**Figure 6:**
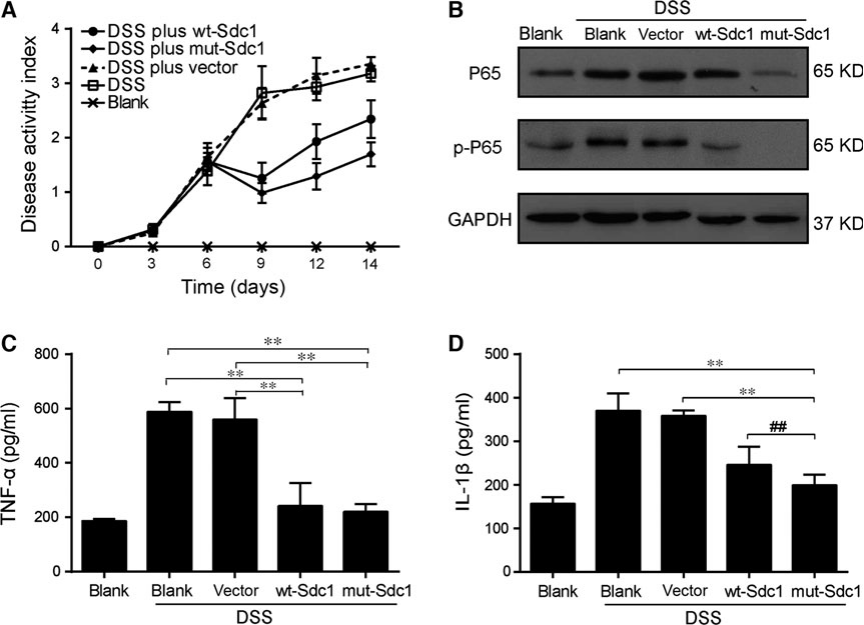
Effects of cell surface SDC1 on activity of colitis in mice. (**A**) DAI value during the course of DSS‐induced colitis. (**B**) Activities of NF‐κB pathway in colonic mucosa of mice. Secreted TNF‐α (**C**) and IL‐1β **(D)** in serum of mice. Values represent mean ± S.E.M (*n* = 3) and were analysed by Duncan's multiple range test for multiple comparison in anova (**P < 0.01. # significance between wt‐SDC1 and mut‐SDC1, ##*P* < 0.01).

To further investigate the effects of SDC1 on intestinal inflammation *in vivo*, we introduced the mouse model of DSS‐induced colitis. Immunohistochemical staining showed that DNA transfection *in vivo* successfully enhanced SDC1 expression in colon mucosa in the DSS plus wt‐SDC1 and DSS plus mut‐SDC1 groups, especially in mut‐SDC1‐treated group, since partial SDC1 was cut from the epithelial by DSS in wt‐SDC1‐treated group (Fig. 5, the bottom row). Histological images of the colon from the DSS‐treated mice showed destruction of the epithelial architecture, impaired crypts and glands, multifocal shallow ulcers and intense inflammatory cell infiltration in the lamina propria; however, fewer of these lesions were seen in the SDC1‐transfected group than in vector‐transfected mice (Fig. 5, the top row). The control mice without DSS treatment had normal bw, colons and stools. Diarrhoea and guaiac positivity was noted in the DSS and the DSS plus vector group, starting on day 3 following DSS treatment; gross bleeding was detected starting on day 6. Weight loss, shorter colons and gross ulceration was also recorded. In contrast, the colitis symptoms were substantially relieved in the DSS plus wt‐SDC1 and DSS plus mut‐SDC1 groups. Consistently, DAI scores were significantly higher in mice 6 days after DSS treatment; while the increased score was suppressed in DSS plus wt‐SDC1 and DSS plus mut‐SDC1 groups (*P* < 0.05, Fig. 6A). Moreover, the improved symptoms of colitis, and the reduced DAI scores were more marked in the DSS plus mut‐SDC1 group than in the DSS plus wt‐SDC1group. These results indicate that SDC1 mitigates disease activity as well as the tissue damage associated with DSS‐induced colitis, and suppression of ectodomain shedding further improves these effects.

### Cell surface‐anchored SDC1 inhibited inflammatory pathway activation and cytokine secretion during DSS‐induced colitis

Higher levels of P65 and p‐P65 were observed in DSS‐induced inflammatory tissues compared with normal tissues, but this increase was markedly attenuated in tissues with supplemented Sdc1 by DNA transfection (Fig. 6B). Similarly, serum levels of TNF‐α and IL‐1β were significantly up‐regulated after DSS administration, compared with the control group. The increases in secretion were lessened when wt‐SDC1 transfection or mut‐SDC1 transfection was introduced (*P* < 0.05, Fig. 6C and D). Moreover, for IL‐1β, the reductive extents were more notable in the DSS plus mut‐SDC1 group (*P* < 0.05, Fig. 6D). These data indicate that SDC1 negatively regulates the pro‐inflammatory cytokine secretion, and this influence may be primarily dependent on the suppression of ectodomain shedding.

## Discussion

Our study describes a mechanism in which three components, a common disease (UC), a cell‐bound proteoglycan with shedding‐resistance (SDC1) and an inflammatory response (neutrophil transmigration), act coordinately to function. We successfully constructed a model in which SDC1 is not shed off from the cell surface upon PMA stimuli, which will facilitate further study of this topic. And the results document a significant correlation between SDC1 shedding and the regulation of inflammation in colitis patients’ samples and intestinal epithelial cells. It is also showed that SDC1, especially when anchored on the cell surface, inactivates several canonical inflammatory pathways, down‐regulates pro‐inflammatory cytokines and inhibits neutrophil migration induced by CXCL‐1. These results were further confirmed in a mouse model of DSS‐induced colitis. To sum up, these findings indicate that the cell surface‐anchored SDC1 alleviates the inflammatory response by inhibiting inflammatory pathways (such as NF‐κB) and pro‐inflammatory cytokines (such as TNF‐α) to a certain extent.

These above results are consistent with those found in patients with Crohn's disease (CD), which demonstrated the decreased levels of SDC1 in the intestinal mucosa and the increased levels of its ectodomain in the serum 25. However, our results are different from a recent study performed by Israeli researchers, which showed elevated levels of soluble SDC‐1 in CD patients, but no difference between UC patients and healthy controls 26. The causes of the differentiated outcomes between Israeli patients and our study were unknown yet. Both studies recruited a small number of patients, and the inconsistency of disease activity, even race, probably had some effects.

The intestine is a unique tissue, where an elaborate and harmonious balance is maintained that has often been referred to as a state of controlled inflammation. In the event where this balance is lost, diseases, such as inflammatory bowel disease (IBD), coeliac disease or food allergy, would come up 27. Recently, accumulated evidence highlights the significance of SDC1 in this balance. Syndecan‐1 is essential in maintaining intestinal epithelial barrier function, specific loss of SDC1 might wreck the natural barrier, causing far more susceptibility to protein‐loss enteropathy and bacterial translocation 7, 28. Recent studies have also showed the interactions between SDC1 and tight junction protein. Syndecan‐1 co‐expresses with Claudin‐2, ZO‐1 and Occludin decrease synchronously with SDC1 destruction 29, 30. Besides, pathological destrction of SDC1 would disrupt internal immune system. We have confirmed the protective role of SDC1 in intestinal inflammatory responses. Previous studies also showed *Pseudomonas aeruginosa* activated the ectodomain shedding of SDC1, but such SDC1 shedding promotes *P. aeruginosa* pathogenesis in mouse models of lung and burned skin infection 31. It is well‐known that CXCL‐1 could induce inflammatory events *via* neutrophil transmigration 32. Syndecan‐1 also contributes to neutrophil chemotaxis: shed and exogenous SDC1 ectodomain induces neutrophil chemotaxis, inhibits epithelial wound healing and promotes fibrogenesis in a mouse model of idiopathic pulmonary fibrosis 33. In mouse *S. aureus* corneal infection, the ectodomain of SDC1 could inhibit *S. aureus* killing by antimicrobial factors secreted by degranulated neutrophils, but does not affect intracellular phagocytic killing by neutrophils, indicating that SDC1 has a close association with the factors secreted by neutrophils 34. Syndecan‐1‐null endotoxemic mice showed a deficiency in the removal of CXCL‐1 in tissues, conversely, SDC1 shedding facilitated the clearance of CXCL‐1 and resolution of neutrophilic inflammation in an HS‐dependent manner 16. However, the relationship between SDC1 and CXCL‐1‐dependent neutrophil migration in intestinal inflammation had rarely been studied. Our findings show that high levels of CXCL‐1 recruit additional neutrophils and aggravate intestinal injury; this inflammatory response can be abolished by SDC1, especially by its cell‐bound ectodomain. When SDC1 shedding was suppressed, CXCL‐1 secreted by intestinal cells would significantly down‐regulate and neutrophil migration induced by conditioned growth media from these cells also decreased. Moreover, these reductive extents are paralleled with inactivation of NF‐κB pathway and down‐regulation of cytokines including TNF‐α, IL‐1β, IL‐6 and IL‐8.

Pathways such as NF‐κB is recognized as critical regulators of epithelial tissue homoeostasis and pathogenesis of inflammatory diseases. Mucosal inflammation in patients with IBD, and in experimental models of intestinal inflammation, is accompanied by elevated levels of activated members of the NF‐κB family, particularly P65, P50 and c‐Rel 35, 36. And deletion of P65 attenuated LPS‐induced cytokine production, CCL11 expression in myeloid cells, as well as inflammation and histopathology in mice 37. In response to LPS or DSS‐induced SDC1 shedding, intestinal epithelial cells showed P65 and phosphorylated P65, activated NF‐κB. More importantly, when its ectodomain shedding was inhibited, the anchored SDC1 significantly attenuated these inflammatory effects. Furthermore, we extended this network by showing the synchronously induction of pro‐inflammatory cytokines, including TNF‐α, IL‐1β, IL‐6 and IL‐8.

The inflammation‐associated network involves various factors which interact with each other to form a complicated feedback loop to function. For example, TNF‐α, IL‐1β, IL‐6, IL‐8 and CCR2 are believed to be target genes of NF‐κB through DNA‐binding to the corresponding promoter 38, 39. Otherwise, TNF‐α and IL‐1β were highly pro‐inflammatory agents, accelerating NF‐κB activation directly *via* their ability to induce expression of IL‐6 40, 41. Furthermore, NF‐kB/p65 interacts with Stat3. Stat3 up‐regulated NF‐κB nuclear retention and enhanced NF‐κB/P65 acetylation *via* serine and tyrosine phosphorylation as well as the DNA‐binding domain of Stat3 42. Our results are in agreement with these previous findings, although the signalling crosstalk within the cell surface SDC1‐associated complex is not clearly elucidated. Thus, further investigation is needed.

The shedding of SDC1 ectomain and neutrophil transmigration in UC might provide a new rationale for disease incidence and therapeutic approach. More attention should be paid to investigate their practical clinical values. Our group has already confirmed the protective role of low‐molecular weight heparin, which is an analogue of SDC1 and can inhibit SDC1 shedding and consequently alleviate inflammation in DSS‐induced mice colitis 43. In accordance with results of small trials which heparins benefits the SDC1 integrity in active glucocorticosteroid refractory in IBD 44, 45. However, patients should take a risk of possible adverse events such as serious bleeding. So a modified heparin or sulphated oligosaccharide may be a breakthrough of clinical application 46, 47.

In conclusion, we have found a protective role of SDC1 anchored on the cell surface in inhibiting intestinal inflammation and neutrophil transmigration. This observation indicates the potential value and important insights of the therapeutic use of SDC1 for improving intestinal inflammation. Suppression of SDC1 shedding in intestinal epithelial cells plays an anti‐inflammatory role, ameliorates colitis and thus is helpful for this disorder treatment.

## Conflicts of interest

All the authors disclose no conflicts.

## Author contribution

ZQW, YZ and JL conducted the experiments; ZQW and YC wrote the manuscript; JDW, PWP and YC designed and supervised the research; JXF, JLi, SHZ and TZ contributed to materials and *in vitro* experiments.
