# Corrigendum

**DOI:** 10.1111/jcmm.13151

**Published:** 2017-03-10

In Yan Zhang *et al*. 1, page 23, the grant number supported by the National Natural Science Foundation of China was incorrect and it should have been “Ye Chen, no. 81570480”.

The authors declared no conflict of interest and wish to apologize for any misunderstanding or inconvenience this may caused.
